# Systemic Analysis of the Prognosis-Associated Alternative Polyadenylation Events in Breast Cancer

**DOI:** 10.3389/fgene.2020.590770

**Published:** 2020-11-03

**Authors:** Yi Zhang, Yuzhi Wang, Chengwen Li, Tianhua Jiang

**Affiliations:** ^1^Department of Blood Transfusion, People’s Hospital of Deyang City, Deyang, China; ^2^Department of Laboratory Medicine, People’s Hospital of Deyang City, Deyang, China; ^3^School of Basic Medical Science, Southwest Medical University, Luzhou, China

**Keywords:** alternative polyadenylation events, breast cancer, overall survival, relapse-free survival, signatures

## Abstract

Alternative polyadenylation (APA) is a post-translational modification that occurs during mRNA maturation in humans. Studies suggested that abnormal APA events are associated with the genesis and progression of malignant tumors. Here, we aimed to comprehensively evaluate the prognostic value of APA events involved in breast cancer (BC). Both APA events and clinical information for BC patients were downloaded from The Cancer Genome Atlas (TCGA) database to identify prognosis-related APA events in BC. A total of 462 APA events and 374 APA events were shown to be significantly related to overall survival (OS) and relapse-free survival (RFS), respectively, of BC patients. The TCGA set was randomly divided into a training and a test set. Key prognosis-related APA events were selected by LASSO regression to build prediction signatures for OS and RFS by multivariate Cox regression analysis in the training, test, and whole set. BC patients were stratified into high-risk and low-risk groups based on median risk scores. Kaplan–Meier survival analysis demonstrated that low-risk groups had better OS and RFS than high-risk groups in all three sets. The time-dependent receiver operating characteristic (ROC) curves showed that our signatures had a good predictive ability for survival and recurrence for BC patients in all three sets. The independent prognostic indicators-based nomogram model had excellent performance and considerable net benefit for predicting the OS and RFS in BC. A PPI network was constructed between key prognosis and core regulators associated with APA, consisting of 48 nodes and 244 edges. Functional enrichment analysis also revealed their association with RNA processing and RNA synthesis. Collectively, our data indicate that prognostic signatures based on APA events may be powerful prognostic predictors for OS and RFS in BC.

## Introduction

Breast cancer (BC) is the most common malignant disease worldwide and the second cause of cancer-related deaths in women. In 2019, there were approximately 268,600 newly diagnosed BC cases worldwide and over 40,000 deaths in 2019 (Siegel et al., 2019). Even though remarkable advancements in therapeutic modalities have been made over the years, such as precise surgery, chemotherapy, radiotherapy, hormonal therapy, and molecular targeting therapy, the average 5-year survival rate for BC is still unsatisfactory due to recurrence and metastasis of advanced-stage tumors (Emens, 2018; Ferlay et al., 2019). Therefore, early diagnosis is key for improving outcomes and survival, as well as the cornerstone of successful treatment. Some traditional tumor markers are widely used to assess screening, treatment responses, prognosis, and recurrence. The mammography, CA153 and CEA, are commonly used, but they lack adequate sensitivity and specificity (Duffy et al., 2010; Weigel and Dowsett, 2010; Bleyer and Welch, 2012). Therefore, more effective biomarkers are needed to evaluate prognosis and occurrence of BC. Such markers can be identified through the elucidation of the molecular mechanisms underlying BC development and progression.

Alternative polyadenylation (APA), a way of processing and modification targeting at 3′ ends of pre-mRNA during eukaryotic mRNA maturation, is an important post-transcriptional regulatory mechanism (Elkon et al., 2013). The 3′ untranslated region (UTR) of mRNAs contains key RNA regulatory elements (Park et al., 2018). APA influences mRNA stability, translation efficiency, and localization by regulating the length of the 3′ UTR length (Mayr, 2016; Tian and Manley, 2017). A majority of human transcripts contain multiple polyadenylation sites (PAS) as shown by transcriptome-wide studies, demonstrating that APA is widespread throughout the genome (Chen and Shyu, 2017). Given the critical roles of APA plays in the biological process, it is also closely related to tumor occurrence and development by regulating transcription and translation of oncogenes and tumor suppressor genes (Sandberg et al., 2008; Mayr and Bartel, 2009). For example, in glioblastoma, the APA regulator CFIm25 upregulates oncogenes to enhance tumorigenic properties and tumor size by shortening the 3′ UTR length (Masamha et al., 2014). In mantle cell lymphoma, the shortened 3′ UTR length significantly reduces miRNA inhibition and elevates Cyclin D1, which increases lymphoma cell proliferation and decreases overall survival (OS). Although the biological significance of APA is widely recognized, its clinical application as a biomarker and therapeutic target for cancers has not been fully evaluated. Therefore, a systematic understanding of APA events may provide new insights into the prognosis and occurrence of certain cancers.

A single-cancer APA analysis with a large patient cohort has not been performed due to funding, testing costs, and patient source constraints. Fortunately, with the explosive development of sequencing and information technology, a large number of patient test results and information have been uploaded and stored in public databases. These data provide abundant resources for researchers to elucidate therapeutic targets for diseases from the molecular perspective. Here, we systematically analyzed the prognostic roles of APA events in BC samples extracted from The Cancer Genome Atlas (TCGA) and constructed prognostic risk score signatures to predict BC prognosis. In addition, we explored the function of APA events and APA regulators as well as established a network emphasizing their relationships, which might shed new light on the contributions of APA events in tumor development and progression.

## Materials and Methods

### Data Collection and Preprocessing

The APA events data for BC were obtained from the UCSC Xena Browser^1^ and corresponding clinical information were downloaded from the TCGA data portal^2^. To mitigate the impact of individuals who passed away or quit the study as a result of factors not associated with BC, patients with an OS time or relapse-free survival (RFS) time less than 30 days were excluded from the study. The percentage of distal polyA site usage index (PDUI) value was an intuitive ratio (ranging from 0 to 1) used to quantify an APA events. The following criteria were used to select APA events: average PDUI values > 0.05 and standard deviation of PDUI values > 0.01. Afterward, missing PDUI values were imputed with the K-nearest neighbor algorithm, as implemented in the “impute” package in R software (version 4.00).

### Identification of Prognosis-Associated APA Events and Prognostic Model Construction

Univariate Cox regression analysis was applied to identify the OS-related APA events and RFS-related APA events, and the top 20 most significant OS-related APA events and RFS-related APA events were selected for the next analysis. LASSO regression analysis at 10-fold cross-validation was used to further narrow down the prognosis-related APA events and prevent model over-fitting (Tibshirani, 1997). Subsequently, the whole cohort was randomly assigned to the training and the test sets at a 7:3 ratio. Multivariate Cox proportional hazards regression was performed to construct prognostic models for OS and RFS in the three sets. The risk score for each patient was calculated using the following formula: risk score value = EXP1 × β1 + EXP2 × β2… + EXPx × βx, where EXP stands for each APA event value and β stands for the regression coefficient from the model. All patients were divided into high-risk and low-risk groups based on the median risk scores. A log-rank test was carried out to compare the differential survival between low-risk and high-risk subgroups. The time-dependent receiver operating characteristic (ROC) curves were adopted to assess the predictive capacity of the prognostic models.

### Cox Regression Analysis and Nomogram Analysis

Univariate Cox regression analysis was used to identify clinicopathological characteristics and signatures correlating with BC prognosis. Clinicopathological characteristics included age, pM, pN, pT, stage, estrogen receptor (ER), progesterone receptor (PR), and human epidermal growth factor receptor 2 (HER2). Subsequently, multivariate Cox regression analysis was performed on all variables showing statistical significance in univariate Cox analysis to evaluate the independent prognostic factors for BC patients. A Chi-squared test was used to investigate the relationship between the signatures and clinicopathologic characteristics. Besides, the independent prognostic factors identified from analyses were included to build nomogram models. Calibration plots and decision curve analysis (DCA) were conducted to examine prognostic accuracy and clinical benefit for the nomogram models.

### Functional Enrichment Analysis

The 22 core regulators (CRs) for APA were collected based on previous reports (Shi et al., 2009). The intersection between OS-related APA events and RFS-related APA events was selected as a prognosis hub for APA events. We used the “clusterProfiler” package in R to perform Gene Ontology (GO) and Kyoto Encyclopedia of Genes and Genomes (KEGG) pathway enrichment analysis for the hub APA events and CRs. The GO term enrichment analysis included three categories: molecular function (MF), cellular component (CC), and biological process (BP). A *P* value < 0.05 was used to determine statistical significance. To explore interactions, a protein-to-protein (PPI) network was established based on the Search Tool for the Retrieval of Interacting Genes (STRING) database^3^ and visualized using Cytoscape software (version 3.7.0).

## Results

### Screening Key Prognosis-Associated APA Events

**FIGURE 1:**
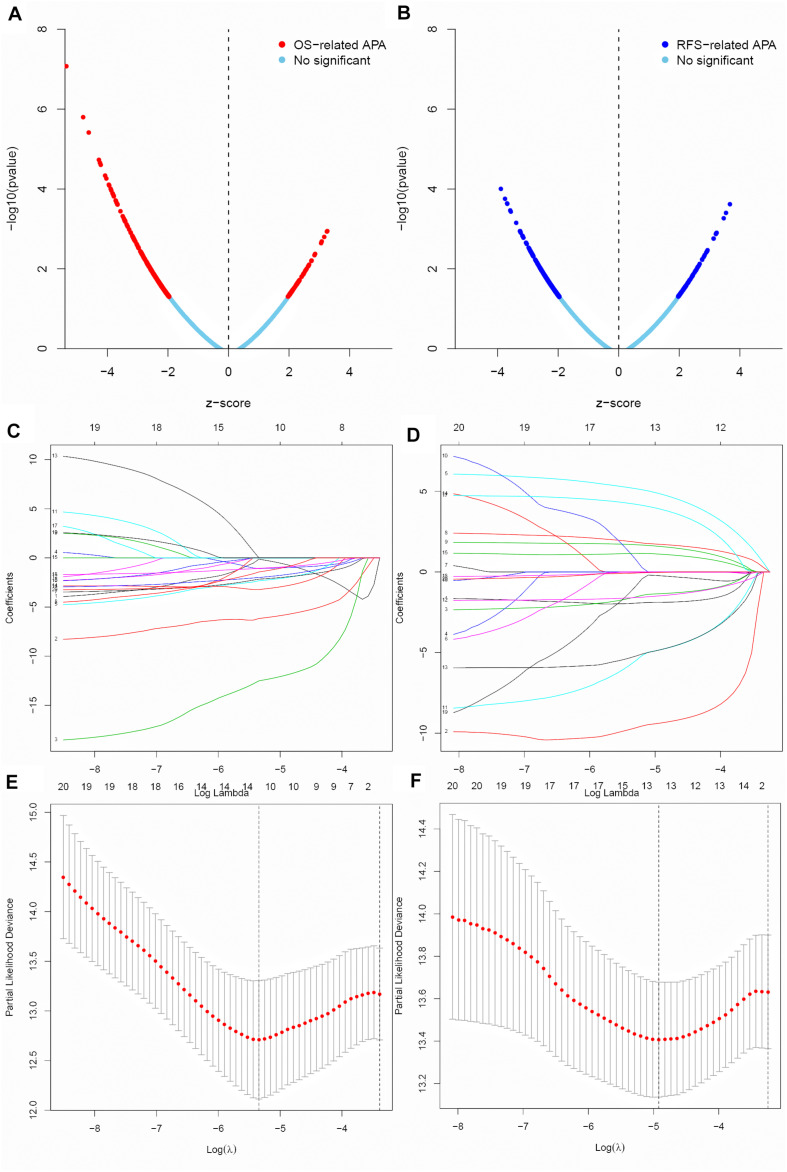
Screen of the key prognosis-related APA events. **(A,B)** Volcano plot of OS-related APA events and RFS-related APA events. **(C,D)** Ten-fold cross-validation for tuning parameter selection in the LASSO regression for OS and RFS. **(E,F)** LASSO coefficient profiles of top 20 prognostic APA events for OS and RFS.

A total of 9869 APA events in 9206 genes were identified in the BC patients, indicating that the great majority of genes have a unique APA event. Univariate Cox regression analysis was performed to screen the prognosis-associated APA events for BC patients. In totally, 462 OS-related APA events (Figure 1A) and 374 RFS-related APA events (Figure 1B) were detected in BC. The top 20 most significant OS-related APA events and RFS-related APA events were selected for further analysis. LASSO regression was used to identify key prognosis-associated APA events for OS (Figures 1C,E) and RFS (Figures 1D,F) in BC.

### Construction of APA-Based Prognostic Signatures

**FIGURE 2:**
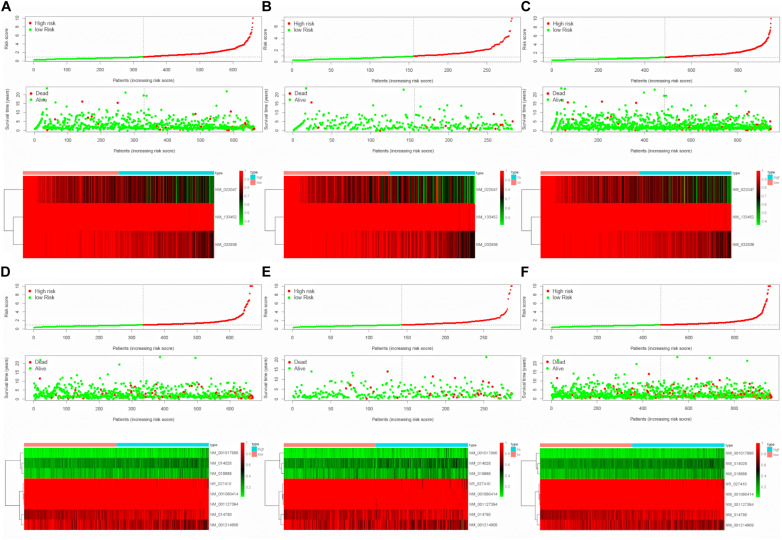
Construction of multivariate Cox proportional hazards regression models. **(A–C)** Distribution of risk score, status, and APA events heatmap for OS in the training set, test set, and whole set. **(D–F)** Distribution of risk score, status and APA events heatmap for RFS in the training, test and whole sets.

**FIGURE 3:**
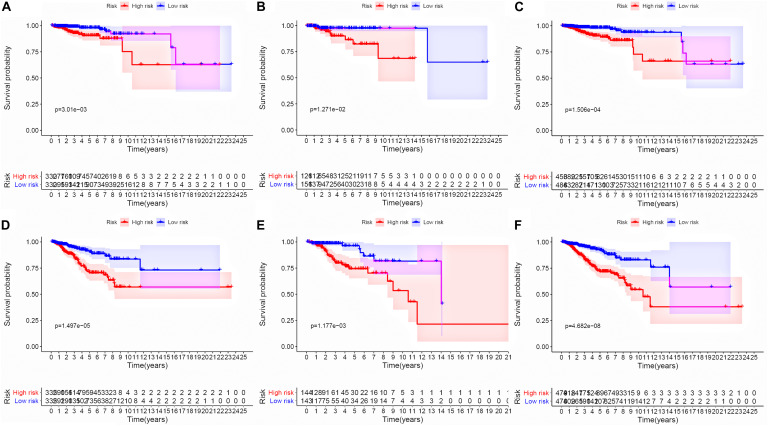
Kaplan–Meier survival analysis between high groups and low groups. **(A–C)** Survival curves of BC patients from high-risk groups and low-risk groups in training, test, and whole sets based on the signature risk score for OS. **(D–F)** Survival curves of BC patients from high-risk groups and low-risk groups in training, test, and whole sets based on the signature risk score for RFS.

**FIGURE 4:**
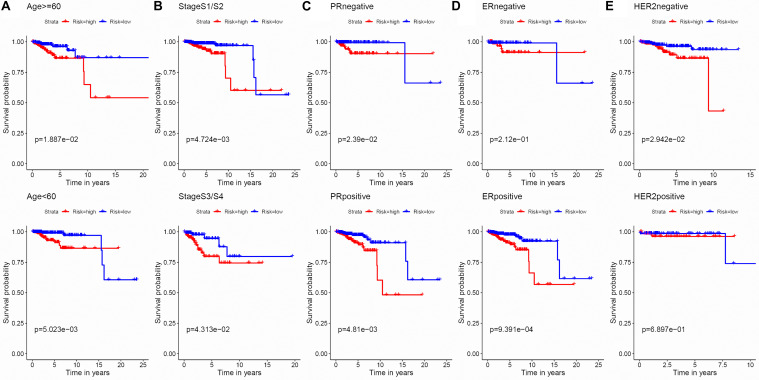
Kaplan–Meier survival analysis of the 3-APA-event risk score level for OS in different subgroups. **(A)** Age < 60, Age ≥ 60. **(B)** Stage 1/2, Stage 3/4. **(C)** PR negative, PR positive. **(D)** ER negative, ER positive. **(E)** HER2 negative, HER2 positive.

**FIGURE 5:**
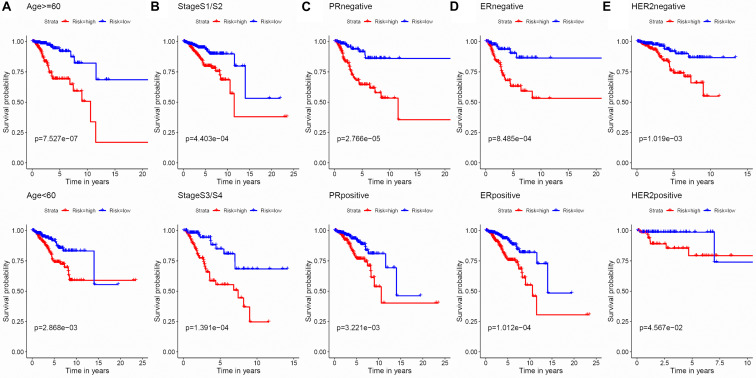
Kaplan–Meier survival analysis of the 8-APA-event risk score level for RFS in different subgroups. **(A)** Age < 60, Age ≥ 60. **(B)** Stage 1/2, Stage 3/4. **(C)** PR negative, PR positive. **(D)** ER negative, ER positive. **(E)** HER2 negative, HER2 positive.

**FIGURE 6:**
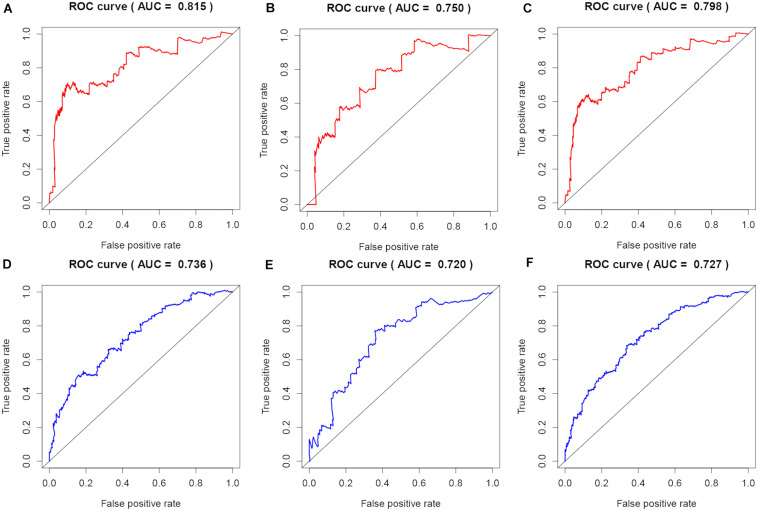
Evaluation of predicting prognostic ability for models. **(A–C)** Time-dependent ROC curves of the signature at 5 years for OS in the training, test, and whole sets. **(D–F)** Time-dependent ROC curves of the signature at 5 years for RFS in the training, test, and whole sets.

To evaluate the stability and applicability of the models, we randomly assigned the whole cohorts into training and test sets. For OS, BC patients (*n* = 942) were randomly divided into a training set (*n* = 660) and a test set (*n* = 282) at a 7:3 ratio. Likewise, BC samples (*n* = 957) were randomly divided into a training set (*n* = 670) and a test set (*n* = 287) at the same ratio for RFS. Next, three APA events (NM_133452, NM_032936, and NM_022047) and eight APA events (NR_027410, NM_014028, NM_014780, NM_018688, NM_001080414, NM_001214909, NM_001127394, and NM_001017986) were included to build the multivariate Cox proportional hazards regression models for OS (Figure 2A) and RFS (Figure 2D) in the training sets, respectively. Meanwhile, the same models for OS (Figures 2B,C) and RFS (Figures 2E,F) were also established in the test and the whole sets. BC patients were split into high-risk groups and low-risk groups according to the median risk scores. Kaplan–Meier survival plots demonstrated that the high-risk groups exhibited shorter OS and RFS compared with low-risk groups in the training (Figures 3A,D), test (Figures 3B,E), and the whole sets (Figures 3C,F). Patients with different ages, stages, PR status, ER status, and Her2 status were stratified based on the risk levels, and survival curves were generated for OS and RFS. Patients in low-risk groups showed better OS than the patients in the high-risk groups at age < 60, age ≥ 60, stage 1/2, stage 3/4, PR negative, PR positive, ER positive, and HER2 negative (Figure 4). At the same time, the patients in low-risk groups showed better RFS than the patients in high-risk groups at age < 60, age ≥ 60, stage 1/2, stage 3/4, PR negative, PR positive, ER negative, ER positive, HER2 negative, and HER2 positive (Figure 5). Also, time-dependent ROC curves indicated that area under curves (AUCs) of the OS signature for 5 years in the training, test, and whole sets were 0.815, 0.750, and 0.798, respectively (Figures 6A–C). As for RFS, the AUCs based on the signature for 5 years in the training, test, and whole sets were 0.736, 0.720, and 0.727, respectively (Figures 6D–F). These results revealed that two signatures showed powerful prognostic ability for BC.

### Independence of Prognostic Factors and Establishment of Nomograms

**FIGURE 7:**
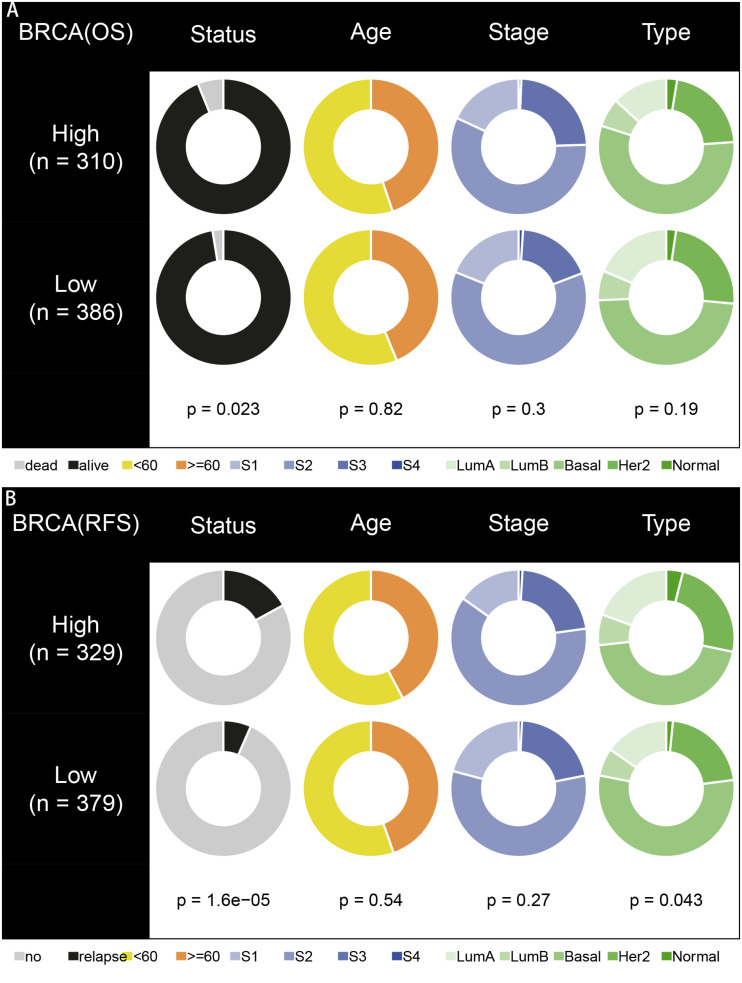
Correlation analysis of signatures with clinicopathological characteristics in the whole set. **(A,B)** Pie charts showing Chi-squared test of clinicopathologic factors for OS and RFS in different groups.

**FIGURE 8:**
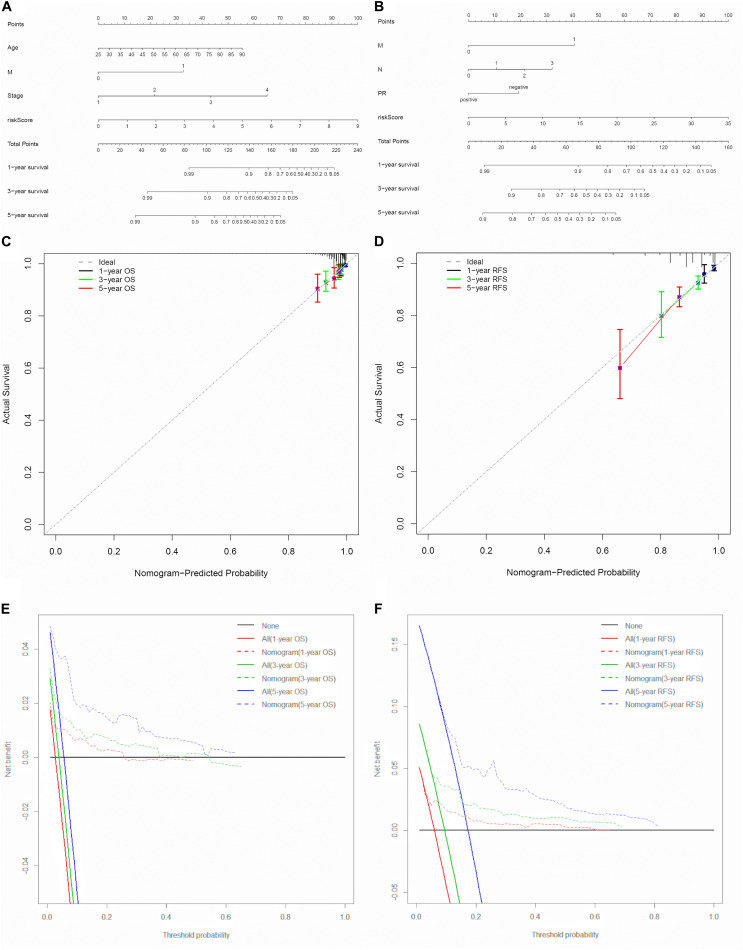
Nomogram models for the prediction of BC prognosis in the whole set. **(A,B)** Nomograms for predicting probability of OS and PFS at 1, 3, and 5 years. **(C,D)** Calibration curves of the nomograms at 1, 3, and 5 years for OS and RFS. **(E,F)** Decision curves of the nomogram at 1, 3, and 5 years for OS and RFS.

To explore the prognostic value of the signatures and clinicopathological variables for patients with BC, Cox regression analysis was performed for the whole set. Univariate Cox regression analysis found that age, pM, pN, pT, stage, and signature were obviously related to OS (Table 1) and that pM, pN, pT, stage, PR, and signature were related to RFS (Table 2) (*P* < 0.05). Multivariate Cox regression analysis was performed for these prognosis-related indicators. The results showed that age, pM, stage, and signature were remarkably related to OS (Table 1) and that pM, pN, PR, and signature were remarkably related to RFS (Table 2), which may be regarded as the independent prognostic indicators of BC survival and recurrence. We also performed a Chi-squared test to assess the association between signatures and main clinical and pathologic features in the whole set. As shown in Figure 7, the two groups showed significant differences in two characteristics, including status and type, in the whole sets for OS and RFS. The high-risk group was especially more likely to exhibit the basal molecular types, while the low-risk group was more likely to exhibit HER2 and lumA molecular types for OS. For RFS, the high-risk group was more likely to exhibit normal and lumA molecular types, while the low-risk group was more likely to exhibit basal molecular types. Next, in order to provide a quantitative method for predicting the prognosis of BC patients in different years, we established nomograms models based on the independent prognostic indicators for OS (Figure 8A) and RFS (Figure 8B) in the whole set. Hence, the OS and RFS survival probability for each BC patient in 1, 3, and 5 years could be quantified by total points in the nomograms. Moreover, calibration curves for OS (Figure 8C) and RFS (Figure 8D) in different years exhibited strong concordance between prediction and observation. The clinical use of the nomogram was evaluated by the DCA. The decision curve showed that the nomogram provided greater net benefit than the “treat all” or “treat none” situations regarding OS (Figure 8E) and RFS (Figure 8F) probability in different years.

**TABLE 1:** COX regression analysis of the 3-APA-event signature with OS.

**Parameters**	**Univariate COX**		**Multivariate COX**	
	**HR (95% CI)**	***P* value**	**HR (95% CI)**	***P* value**
Age	1.038 (1.103, 1.064)	0.002	1.037 (1.009, 1.066)	0.009
PR	1.409 (0.668, 2.884)	0.348		
ER	1.654 (0.693, 3.946)	0.257		
HER2	0.791 (0.269, 2.320)	0.669		
Stage	3.276 (2.106, 5.097)	<0.001	3.201 (1.486, 6.895)	0.003
T	1.187 (1.180, 2.460)	0.004	0.798 (0.477, 1.334)	0.389
M	15.831 (7.395, 33.909)	<0.001	3.575 (1.172, 10.902)	0.025
N	1.494 (1.082, 2.062)	0.015	0.928 (0.589, 1.461)	0.747
Risks core	1.244 (1.146, 1.351)	<0.001	1.678 (1.395, 2.018)	<0.001

**TABLE 2:** COX regression analysis of the 8-APA-event signature with RFS.

**Parameters**	**Univariate COX**		**Multivariate COX**	
	**HR (95% CI)**	***P* value**	**HR (95% CI)**	***P* value**
Age	1.002 (0.987, 1.017)	0.814		
PR	0.558 (0.380, 0.819)	0.003	0.439 (0.236, 0.819)	0.010
ER	0.604 (0.402,0.906)	0.015	0.914 (0.481, 1.739)	0.785
HER2	0.854 (0.415, 1.760)	0.669		
Stage	2.203 (1.666, 2.912)	<0.001	1.038 (0.559, 1.928)	0.905
T	1.647 (1.291, 2.102)	<0.001	1.227 (0.793, 1.898)	0.357
M	7.838 (3.787, 16.219)	<0.001	5.095 (1.823, 14.243)	0.002
N	1.685 (1.390, 2.041)	<0.001	1.462 (1.045, 2.047)	0.027
Risks core	1.173 (1.134, 1.214)	<0.001	1.106 (1.052, 1.163)	<0.001

### GO Functional Enrichment and KEGG Pathway Analysis

**FIGURE 9:**
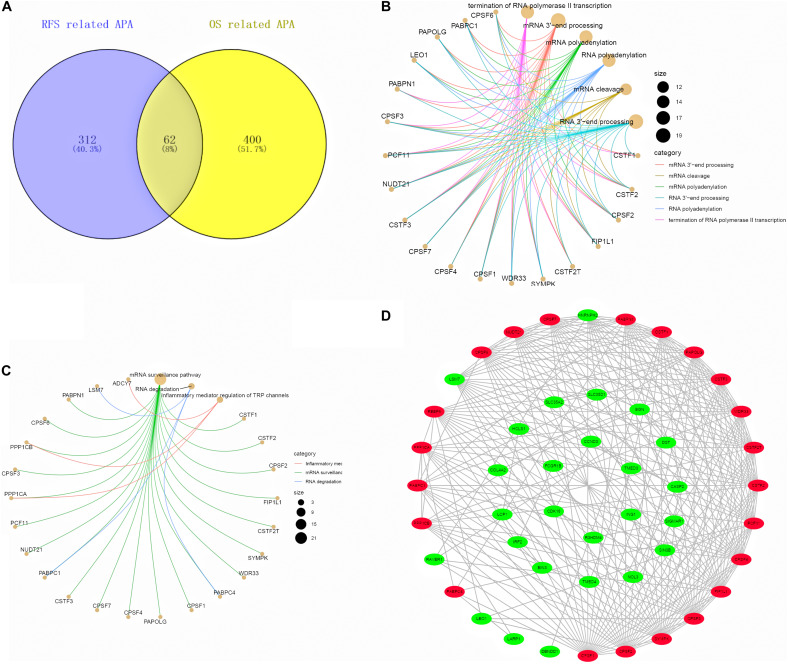
Interaction network between the hub APA genes and CRs. **(A)** Venn diagram of the hub APA events in RFS-related APA events and OS-related APA events. **(B,C)** GO and KEGG analysis of the hub APA genes and CRs. **(D)** PPI network of the hub APA genes and CRs.

As a result, the 62 hub prognosis APA events were obtained through the Venn diagram (Figure 9A). Subsequently, we performed functional enrichment analysis on hub prognosis APA genes and CRs. GO analysis suggested that these genes were mainly enriched in mRNA 3′ end processing, mRNA cleavage, mRNA polyadenylation, RNA 3′ end processing, RNA polyadenylation, and termination of RNA polymerase II transcription (Figure 9B). Based on the KEGG analysis, these genes were notably enriched in inflammatory mediator regulation of TRP channels, mRNA surveillance pathway, and RNA degradation (Figure 9C). To identify the potential interactions between these genes, we built a PPI network using the STRING database and Cytoscape software, which consists of 48 nodes and 244 edges (Figure 9D).

## Discussion

Alternative polyadenylation is highly prevalent in eukaryotic cells. Hoque et al. (2013) reported that at least 70% of human genes display APA events. APA is an important mechanism regulating gene expression in several ways. Thus, dysregulated APA may result in abnormal protein function, potentially leading to disease states. It has been reported that tumor samples have global shortening of APA compared with normal samples, which may be potential targets for cancer patients (Sandberg et al., 2008; Mayr and Bartel, 2009; Ichinose et al., 2014). In recent years, there have been more researches determining the biological functions of APA events in BC. For example, (Yan et al., 2018) found APA-mediated shortening of the Ki-67 3′ UTR enhanced mRNA stability and improved translation efficiency for promoting BC cell proliferation. Up-regulated microRNA degrades long isoforms of target genes to reduced long and short isoforms in cells, which is a difference when comparing normal tissues to BC tissues (Liaw et al., 2013). With a focus on the prognostic value of APA events, (Wang et al., 2016) generated the 3′ UTR landscape based on filtered multiple microarrays, and they constructed a novel 3′ UTR-based classifier that considerably improved the prognostic stratification of patients diagnosed with triple-negative BC. However, a systematic analysis of prognosis-associated APA events is still lacking.

In the present study, the prognostic value of APA events in BC patients was systematically explored for the first time using the TCGA database. We found that over 95% of genes undergo only one APA event, which indicated that the APA of genes was unique and some might produce a cancer-associated specific protein. We filtered the 462 OS-related APA events in BC patients through univariate Cox regression analysis, as well as 374 APA events for RFS. Among these APA events, the top 20 APA events in each type were selected to identify key APA events for assessing their prognostic value.

It is well known that cancer initiation and progression are complex processes involving multiple molecular mechanisms. Therefore, one single biomarker may not accurately predict the prognosis of patients. As shown by previous studies, combining multiple indicators can improve the accuracy of prediction. For example, (Wang et al., 2020) demonstrated that a six-gene signature associated with TMB acts as a biological marker and improves the prognosis prediction for BC patients. Another study showed that a three-long non-coding RNA-based model could be used for the diagnosis of triple-negative BC (Liu et al., 2017). We performed a multivariate Cox regression analysis to build a 3-APA-event signature for OS and an 8-APA-event signature for RFS. The BC patients were divided into high-risk and low-risk groups with remarkable differences through two signatures for OS and RFS in the training set. In addition, the AUC of time-dependent ROC from 5 years OS and RFS for the two signatures reached 0.815 and 0.763, respectively, in the training set. What is more, similar results were observed in the test and whole sets, indicating the robustness and reliability of the two signatures in predicting BC prognosis. Afterward, univariate and multivariate Cox regression analyses were used to evaluate the prognostic values of the signatures and clinicopathological variables for BC patients. As a result, age, pM, stage and the 3-APA-event signature were creditable independent predictors for OS and pM, pN, PR, and the 8-APA-event signature were creditable independent predictors for RFS. In addition, patients in two groups showed different molecular types for OS and RFS. We established nomogram models based on the independent prognostic indicators to provide the quantitative prediction of individuals in 1-, 3-, and 5-year OS and RFS in the whole set. In terms of predictive accuracy, calibration curves revealed a considerable agreement between the actual and predicted probabilities of OS and RFS. Notably, nomograms contributed favorable clinical net benefit to BC patients over a wide range of threshold probabilities. Among these APA genes in the signatures, only a small percentage were reported in the process of BC occurrence and progression. DEF6 was first identified in 2003 and plays multiple roles in the immune system (Gupta et al., 2003). DEF6 is overexpressed in invasive BC and increases the growth and invasiveness of BC cell *in vitro* (Li et al., 2009). Further research found that DEF6 effectively inhibits autophagy of BC cells by directly activating mTORC2 and promoting phosphorylation of Akt and FOXO3a (Chen et al., 2013). CUL7 is one of the important ingredients in E3 ubiquitin ligases. The expression of CUL7 is positively correlated with the malignant BC behavior of BC but negatively correlates with the BC prognosis (Qiu et al., 2018). A recent study has reported that CUL7 may increase trastuzumab resistance in Her2-positive BC cells by degrading Ser phosphorylation of IRS-1 and activating the PI3K/AKT pathway, which is of great significance for selecting the optimal treatment plan for BC patients.

GO and KEGG analysis of APA genes and CRs were performed subsequently to investigate the underlying biological functions and their association with BC. Results indicated that APA genes and CRs were significantly correlated with RNA processing, RNA synthesis, and inflammatory mediator regulation of TRP channels. These findings were consistent with the tumorigenic roles of APA events in the previous studies, which regulated mRNA expression and localization by altering the 3′ UTR sequence of transcripts (Singh et al., 2018). TRP channels are regarded as important factors in tumorigenesis and may provide some promising diagnostic and therapeutic targets (Chen et al., 2014). Also, according to the PPI network, most CRs interacted with key APA genes and may be hub factors. These results may offer research direction on how the prognosis-related APA events participated in the tumor signaling pathways.

However, our study incontrovertibly suffered from some limitations. First, some of the data collected from public databases, such as clinical information, were not comprehensive, which may cause bias and errors. Second, we only performed internal scientific verification from a single cohort but lacked external data validation and prospective studies. Finally, both *in vitro* and *in vivo* experiments are still required to verify the biological significance of APA events in BC. Nevertheless, this study was the first to present an assessment of the prognostic value of APA events in BC comprehensively and to provide new molecular changes and potential therapeutic targets.

In summary, we have conducted a comprehensive investigation of APA events in BC patients and screened prognosis-related APA events. More importantly, we constructed nomograms combining independent factors with APA events-based signatures for the prediction of survival and recurrence of BC. In addition, the functional connections and interaction network of APA genes and CRs were also established. These results not only provide new opportunities for BC prognostic biomarkers and therapeutic targets but also enrich our understanding of the roles of APA events in BC.

## Data Availability Statement

The datasets presented in this study can be found in online repositories. The names of the repository/repositories and accession number(s) can be found in the article/Supplementary Material.

## Author Contributions

TJ and YZ conceived and designed the study. CL and YW performed the collection and assembly of data. YZ and YW analyzed the data. YZ and YW wrote the manuscript. All authors read and approved the final manuscript.

## Conflict of Interest

The authors declare that the research was conducted in the absence of any commercial or financial relationships that could be construed as a potential conflict of interest.

## Footnotes

1 https://xenabrowser.net/

2 https://portal.gdc.cancer.gov/

3 http://www.string-db.org/
